# Supplementary material for: Burden of Mental and Behavioral Disorders in Colombia, 2022: A Subnational Analysis Based on Disability-Adjusted Life Years
Source: Int J Environ Res Public Health. 2025 Dec 12;22(12):1854. doi: 10.3390/ijerph22121854 (PMC12733028; doi:10.3390/ijerph22121854)
Supplement: Supplementary file 1 [file ijerph-22-01854-s001.zip › Table S3.pdf]

Table S3. Annex Table GATHER.

	Item	Comment for Compliance
1	Define the indicator(s), the population (including age, sex, and geographical data), and the period(s) for which the estimates were produced.	The study used Disability-Adjusted Life Years (DALYs) as the primary indicator, comprising Years Lived with Disability (YLDs) and Years of Life Lost (YLLs).<br><br>The study population included all residents of Colombia in 2022, totaling 51,874,024 individuals distributed across the country’s 32 departments.<br><br>All cases and deaths officially recorded in national health information systems were included, provided they met valid classification criteria under the cause group “Mental and behavioral disorders”, according to the International Classification of Diseases, 10th Revision (ICD-10).
2	State the funding sources for the activity.	This research was funded by the Ministry of Science, Technology, and Innovation of Colombia (MINCIENCIAS) and the University of Los Llanos, through the project titled:<br>*“Development of a Mental Health, Family, and Social Coexistence Observatory for the design of comprehensive strategies, knowledge management, and the formulation of policies, plans, and programs in the Colombian Orinoquía region”* (Code 112291891873, Contract 655 of 2022).<br><br>The funders had no role in the study design, data collection, data analysis, interpretation of results, or the decision to publish the findings.
3	Describe how the data were identified and obtained.	Mortality data were obtained from the Vital Statistics System, contained within the Unique Registry of Affiliation (RUAF) database, while morbidity data were sourced from the Individual Registry of Health Service Provision (RIPS). Both databases are integrated into the Integrated Social Protection Information System (SISPRO).<br><br>Access to these databases is restricted and requires a user account and password issued by the Ministry of Health and Social Protection (MSPS), in accordance with Law 1581 of 2012.<br><br>Mortality estimates were based on the underlying cause of death (UCOD) reported by the MSPS and coded by the National Administrative Department of Statistics (DANE) using the ICD-10 classification.<br><br>Morbidity information was derived from RIPS, which consolidates health care records from 2022. These data are reported by Health Service Provider Institutions (IPS) and validated by Benefit Plan Administration Entities and the MSPS.<br><br>Demographic data were based on the official 2022 census projections from DANE.
4	Specify inclusion and exclusion criteria, and all specific exclusions.	Records of mortality and morbidity related to mental and behavioral disorders among the Colombian population in 2022 were included, provided they contained valid and complete information in the official databases.

Item		Comment for Compliance
		Exclusions comprised mortality records without an underlying cause of death, morbidity records without a registered primary diagnosis, and duplicate entries.<br><br>In addition, the following were excluded: organic mental disorders (including symptomatic forms), mental and behavioral disorders due to tobacco use, disorders resulting from the use of other stimulants (including caffeine), and unspecified mental disorders. Diseases without assigned disability weights in the applied methodology, as well as those grouped under "other" or "unidentified" categories, were also omitted, as they could not be clearly classified or assigned appropriate weights within each group.
5	Provide information on all data sources and their main characteristics. For each source used, include reference information or institutional contact, the represented population, data collection method, year(s) of data collection, sex and age range, diagnostic criteria or measurement method, and sample size, if applicable.	For mortality, data were obtained from the non-fetal deaths database of the RUAF within the Vital Statistics System (EEVV) of Colombia, downloaded from the SISPRO repository. The dataset included death records reported by territorial units for the study year. These records are completed by the attending physician, medical examiner, or non-attending physician through the standardized electronic RUAF platform, following national regulations.<br><br>The variable used to calculate mortality rates was the underlying cause of death (UCOD), reported by the MSPS. The UCOD is defined as "the disease or injury that initiated the train of morbid events leading directly to death, or the circumstances of the accident or violence that produced the fatal injury." The DANE standardizes the diagnostic information in death certificates and codes the UCOD using an algorithm based on the ICD-10 classification system.<br><br>For morbidity, data from individuals who sought health care services in 2022 were obtained from the RIPS database, which consolidates clinical and hospital records generated during patient care in Colombian health facilities. These records are submitted in a standardized format mandated by national regulations. Morbidity data included the main diagnosis or, in the case of injuries, the external cause, as reported by the Health Service Provider Institutions (IPS), and validated by Benefit Plan Administration Entities (EAPBs) and the MSPS.<br><br>Both databases follow a data quality assurance process prior to their publication within SISPRO.<br><br>For both mortality and morbidity datasets, the variables included sex, department, age (0 to maximum recorded years), and the corresponding ICD-10 codes for morbidity or mortality. All available records were included, representing the entire registered population.
6	Mention and describe all types of input data that may be subject to substantial bias	The morbidity and mortality databases used in this study originate from public health information systems, which may be affected by biases related to data quality and completeness. The main potential sources of bias identified were as follows:<br>- Competing risk: The death of an individual with disease A may be attributed to cause B, leading to registration solely under B and an underestimation of the burden of A. This phenomenon may affect cause-specific mortality estimates.<br>- Misclassification: Errors in ICD-10 coding or diagnostic assignment of morbidity or mortality causes, resulting from diagnostic variability, limited test specificity, or inconsistencies in institutional reporting. This represents one of the most relevant potential biases identified.

	Item	Comment for Compliance
		- Access to health care: Registered cases may not represent all events occurring in the community, as they depend on access to the health system, level of care, socioeconomic conditions, cultural factors, and geographical barriers.<br>- Underreporting: The omission of morbidity or mortality cases not captured by the information systems may compromise data completeness and representativeness.<br>Given that the information was obtained from a secondary database consolidated within the SISPRO repository, with no possibility of modifying or revalidating individual records, direct control over these biases was not feasible.
7	List any other input data and specify their source.	To estimate the rates, the population denominators were obtained from the official population projections produced by the National Administrative Department of Statistics (DANE) of Colombia, available at: https://www.dane.gov.co/index.php/estadisticas-por-tema/demografia-y-poblacion/proyecciones-de-poblacion
8	Provide all input data in a file from which data can be efficiently extracted (e.g., preferably in a spreadsheet rather than a PDF), including all relevant metadata specified in item 5. For data that cannot be publicly shared for ethical or legal reasons, such as third-party data, provide the name of the contact person or institution holding the rights.	The databases used in this study are provided as supplementary material.
9	Provide a conceptual overview of the data analysis method; a diagram may be helpful	The analytical approach was based on the Disability-Adjusted Life Year (DALY) metric developed by the Global Burden of Disease (GBD) Study. This metric integrates, within a common time-based unit, both premature mortality and non-fatal morbidity associated with a given disease or group of specific causes.<br><br>DALYs represent the healthy years of life lost in a population, calculated as the sum of Years of Life Lost (YLLs) and Years Lived with Disability (YLDs), adjusted by the severity and duration of the health state.<br><br>This structure enables comparability across diseases with different etiologies and clinical characteristics, as time serves as a common measurement unit. Consequently, DALYs allow for a comprehensive assessment of the burden of disease within a population.<br><br>The total sum of DALYs represents the overall burden of disease in the studied community, making this indicator a versatile tool for health priority setting and public policy guidance.
10	Describe in detail all stages of data analysis, including the mathematical formulas used. This description should include, as applicable, data cleaning, preprocessing, adjustments, weighting of data sources, and the mathematical or statistical models applied.	- Morbidity and mortality data were extracted as pivot tables from the SISPRO system for the year 2022.<br>- The datasets were subsequently converted into individual-level databases using Power Query (Microsoft Excel).<br>- This process included disaggregating grouped cases, and assigning variable categories (sex, age, department, primary diagnosis, or underlying cause of death) to each individual record.<br>- During the data cleaning phase, records with missing underlying cause of death, morbidity cases without a primary diagnosis, and duplicate entries were removed. Additionally, organic mental disorders (including symptomatic forms), mental and behavioral disorders due to tobacco use, disorders related to the use of other stimulants (including caffeine), and unspecified mental disorders were excluded.

	Item	Comment for Compliance
		- Diseases without assigned disability weights and those grouped as “other” or “unidentified” were also excluded, as they could not be clearly classified or assigned a weight within each category.<br>- From the cleaned datasets, DALYs were calculated using the GBD methodology:<br>DALY = YLL + YLD<br><br>- Results were stratified by sex, age group, and department, using official 2022 population projections from DANE as denominators.<br>- Statistical analyses were performed using SPSS software.
11	Describe how the different models were evaluated and how the model(s) used were selected.	The study applied the abbreviated WHO methodology for estimating GBD. This approach was selected for its standardized structure, comparability across causes, and consistency with international burden-of-disease reporting frameworks
12	Report the results of model performance evaluation, if conducted, and the corresponding sensitivity analyses	Not applicable.
13	Describe the methods used to calculate uncertainty in the estimates. Indicate which sources of uncertainty were considered in the analysis and which were not.	Uncertainty in the estimates was assessed using the bootstrap method implemented in SPSS. This statistical resampling technique was applied to obtain empirical distributions of the estimates and their confidence intervals. The procedure follows the standard approach based on the standard error of the sum and the Student’s t distribution.<br><br>1. Calculation of the sum<br>For a variable X_i with n observations:<br>Sum=∑i=1nXi\text{Sum} = \sum_{i=1}^{n} X_iSum=i=1∑nXi<br><br>2. Calculation of the standard error of the sum<br>SPSS first calculates the sample standard deviation (s) and then derives the standard error of the sum from the standard error of the mean, as follows:<br>SEX¯=snSE_{\bar{X}} = \frac{s}{\sqrt{n}}SEX¯=ns SEsum=SEX¯×n=s×nSE_{\text{sum}} = SE_{\bar{X}} \times n = s \times \sqrt{n}SEsum=SEX¯×n=s×n<br><br>3. Calculation of the confidence interval<br>SPSS uses the Student’s t distribution with n – 1 degrees of freedom (for small samples) or the normal distribution (when n is large), and the confidence level specified (by default, 95%).<br>UI=Sum±tα/2, n−1×SEsumaUI = \text{Sum} \pm t_{\alpha/2,\,n-1} \times SE_{\text{sum}}IC=Sum±tα/2,n−1×SEsum
14	Indicate how to access the analytical or statistical source code used to produce the estimates.	Not applicable.

	Item	Comment for Compliance
15	Provide the published estimates in a file format that allows efficient data extraction	The estimates are presented in a complementary annex in .xlsx (Excel) format, which allows efficient data retrieval. This file is available as *Annex 2. Mental and Behavioral Disorders in Colombia, 2022)*
16	Provide a quantitative measure of the uncertainty of the estimates (e.g., confidence intervals).	Uncertainty intervals were generated for the indicator rates.
17	Interpret the results in light of the available evidence. If this is an update of previous estimates, describe the reasons for any changes	The results present the burden of mental and behavioral disorders in Colombia during 2022, disaggregated by sex, age group, and department. The findings reveal significant subnational differences in DALYs.<br>Since this study represents the first recent subnational estimation of the mental health burden in Colombia, it is not a direct update of previous series but rather a complementary approach that provides context-specific evidence useful for public health planning and mental health policy development.
18	Explain the limitations of the estimates. Present an analysis of the model assumptions and the data limitations that affect the interpretation of the estimates.	Although this study provides a robust approximation of the burden of mental and behavioral disorders in Colombia during 2022, it presents inherent limitations related to the use of secondary data sources. The accuracy of the estimates depends on the precision, completeness, and consistency of national records (SISPRO, RUAF, RIPS), which may be affected by diagnostic errors, duplications, or underreporting—particularly for disorders with lower visibility or higher social stigma. Moreover, mortality associated with these conditions tends to be underestimated, as death certificates often prioritize the immediate cause and may not capture the underlying psychiatric disorder. This bias limits the accurate estimation of premature mortality and, consequently, the Years of Life Lost (YLL) component of the burden calculations.<br><br>Finally, gaps in access to mental health services, regional differences in diagnostic capacity across the country, and the exclusion of categories without assigned disability weights in the methodology may affect comparability with other international studies.
