# Supplementary material for: Burden of Mental and Behavioral Disorders in Colombia, 2022: A Subnational Analysis Based on Disability-Adjusted Life Years
Source: Int J Environ Res Public Health. 2025 Dec 12;22(12):1854. doi: 10.3390/ijerph22121854 (PMC12733028; doi:10.3390/ijerph22121854)
Supplement: Supplementary file 1 [file ijerph-22-01854-s001.zip › Table S5.pdf]

Table S5. YLD rate (Years Lived with Disability) by disorders, by department, Colombia 2022.

Department	Mental and behavioral disorders due to alcohol use	Mental and behavioral disorders due to opioids	Mental and behavioral disorders due to cannabinoids	Mental and behavioral disorders due to cocaine	Schizophrenia	Bipolar disorder	Depression	Dysthymia	Anxiety	Bulimia	Anorexia	Conduct disorders in adults	Intellectual disability	Autism	ADHD	Conduct disorders in childhood and adolescence	Depression in childhood and adolescence	Anxiety in childhood and adolescence	Total
Amazonas	13,9 (10 - 17,7)	0,8 (0 - 0)	17,8 (2,6 - 33,1)	13,9 (7,8 - 19,9)	35,3 (21,7 - 49)	6,4 (5,9 - 6,8)	21,4 (15,8 - 27)	0 (0 - 0)	76,2 (47 - 105,3)	0,3 (0 - 0)	0 (0 - 0)	6 (3,4 - 8,7)	8,3 (6,9 - 9,7)	5,9 (2,8 - 9,1)	1 (-0,4 - 2,4)	3,7 (2,7 - 4,7)	0,6 (0,6 - 0,6)	0 (0 - 0)	211,6 (126,9 - 294,1)
Antioquia	11,8 (8,8 - 14,8)	7,6 (4,7 - 10,6)	8,8 (4,3 - 13,4)	13,7 (7,7 - 19,7)	67,4 (43,6 - 91,2)	66 (54,4 - 77,6)	45,6 (37 - 54,2)	1,4 (1 - 1,8)	298,5 (203,8 - 393,2)	0,5 (0,2 - 0,8)	0,8 (0,1 - 1,5)	4,4 (2,6 - 6,2)	8,4 (7,3 - 9,5)	8,9 (2,8 - 15)	5,1 (0,7 - 9,5)	4,8 (2 - 7,6)	0,7 (0,4 - 1)	0,2 (0,1 - 0,4)	554,7 (381,5 - 728,1)
Arauca	3,6 (3,1 - 4,1)	3,7 (2,1 - 5,4)	1,9 (1,1 - 2,6)	1 (0 - 2)	83 (61,9 - 104)	31,9 (27,2 - 36,6)	20,2 (16,1 - 24,3)	2,2 (1,6 - 2,8)	271,1 (181,9 - 360,2)	0,1 (0,1 - 0,1)	0,3 (0 - 0,6)	2 (1,2 - 2,7)	4,9 (4,1 - 5,6)	6,7 (3,4 - 10,1)	3,7 (0,4 - 6,9)	7,8 (4,2 - 11,5)	0,9 (0 - 1,7)	0,2 (0,1 - 0,4)	445,2 (308,5 - 581,8)
Archipiélago de San Andrés, Providencia y Santa Catalina	7,2 (5,5 - 8,9)	1,1 (0 - 0)	4 (2,9 - 5,1)	3,3 (1,6 - 4,9)	140 (113,7 - 166,2)	60 (51,4 - 68,7)	24,4 (18,4 - 30,4)	1,4 (0,8 - 2)	258 (186 - 330,1)	0 (0 - 0)	0 (0 - 0)	2,7 (1,2 - 4,2)	8,8 (7,3 - 10,3)	29,8 (8,6 - 51)	12,2 (0,9 - 23,5)	8,9 (5,5 - 12,3)	0,8 (0,8 - 0,8)	0 (0 - 0)	562,7 (404,4 - 718,5)
Atlántico	2,9 (2,4 - 3,4)	1,9 (1,5 - 2,3)	2 (1,2 - 2,9)	2 (1,5 - 2,4)	118,8 (80,9 - 156,8)	44,2 (37,8 - 50,7)	54,9 (45,7 - 64)	0,3 (0,2 - 0,4)	367,9 (250,3 - 485,4)	0,3 (0 - 0,5)	0,4 (0,2 - 0,6)	2,4 (1,3 - 3,6)	11,8 (9,5 - 14,2)	40 (11,3 - 68,7)	14,7 (2,2 - 27,3)	13,1 (7,7 - 18,6)	1,3 (0,2 - 2,3)	0,5 (0,2 - 0,8)	679,4 (454,2 - 904,8)
Bogotá, D.C.	9,8 (8 - 11,6)	3,7 (2,9 - 4,5)	5,5 (3,3 - 7,6)	2,2 (1,6 - 2,8)	116,7 (80 - 153,4)	44,6 (36,9 - 52,2)	50,9 (43,2 - 58,6)	2,2 (1,5 - 2,9)	324,4 (214,8 - 434)	1 (0,2 - 1,7)	1,3 (0,2 - 2,3)	4,7 (2,8 - 6,6)	12 (10,4 - 13,5)	12,4 (5,3 - 19,5)	5,8 (0,7 - 10,9)	5,1 (3,2 - 7,1)	1,8 (0,3 - 3,2)	0,4 (0,1 - 0,7)	604,5 (415,4 - 793,3)
Bolívar	4 (3,4 - 4,6)	1,4 (1,2 - 1,7)	3,5 (1,8 - 5,1)	4,1 (2,5 - 5,7)	138,1 (98,2 - 178)	82,4 (65,3 - 99,5)	85,8 (64,7 - 106,8)	0,4 (0,2 - 0,6)	348 (236,9 - 459,2)	0,2 (0,1 - 0,3)	0,3 (0,1 - 0,6)	3,5 (2 - 5)	14,1 (10,5 - 17,8)	29 (8,5 - 49,4)	11,6 (3,2 - 20)	14,9 (8,7 - 21,1)	0,7 (0,2 - 1,2)	0,4 (0,1 - 0,7)	742,4 (507,7 - 977,2)
Boyacá	13,1 (10,9 - 15,3)	2,1 (1,8 - 2,3)	2,7 (2 - 3,5)	1,3 (0,9 - 1,7)	95,7 (69,2 - 122,3)	54,8 (46,6 - 63,1)	47 (37 - 56,9)	1,4 (0,9 - 1,9)	364,2 (240,2 - 488,2)	0,8 (0,3 - 1,3)	0,5 (0 - 1,1)	3,1 (2,3 - 3,8)	10,6 (9,3 - 12)	7 (2,7 - 11,4)	2,6 (0,5 - 4,8)	3,4 (1,9 - 4,8)	2,1 (0,2 - 4,1)	0,4 (0,1 - 0,7)	612,9 (426,8 - 799,1)
Caldas	12,1 (9,6 - 14,6)	2,8 (2,1 - 3,4)	12,7 (6,3 - 19,2)	4,3 (2,9 - 5,8)	80,3 (56,6 - 104)	161,5 (137,1 - 186)	139,3 (114,7 - 164)	2 (1,3 - 2,8)	534,2 (354,6 - 713,8)	0,2 (-0,1 - 0,5)	0,7 (0 - 1,3)	5,9 (3,6 - 8,2)	14,2 (11,9 - 16,5)	10,8 (5,2 - 16,4)	11,6 (3,5 - 19,8)	7 (4,7 - 9,3)	2,5 (0,1 - 4,8)	0,4 (0 - 0,8)	1002,6 (714,3 - 1291)
Caquetá	4 (3,4 - 4,7)	2 (1,5 - 2,4)	15 (8 - 21,9)	4,1 (2,9 - 5,4)	28,4 (20,7 - 36,1)	85,2 (69,4 - 101,1)	27,5 (20,3 - 34,7)	0,3 (0,2 - 0,5)	201,9 (131,4 - 272,4)	0,4 (0,2 - 0,5)	0,4 (0,2 - 0,6)	1,9 (1 - 2,7)	11,6 (9,4 - 13,8)	5,8 (0,2 - 11,5)	2,6 (0,4 - 4,9)	1,2 (0,9 - 1,5)	0,4 (0,2 - 0,6)	0,4 (0 - 0,8)	393,1 (270,4 - 516)
Casanare	1,5 (1,3 - 1,8)	0,9 (0,9 - 0,9)	2 (1 - 2,9)	0,9 (0,7 - 1)	37 (26,3 - 47,7)	34,3 (28 - 40,7)	6,6 (5 - 8,1)	0,2 (-0,1 - 0,4)	196,3 (128,9 - 263,7)	0 (0 - 0)	0,1 (-0,5 - 0,8)	2,5 (1,6 - 3,5)	7,2 (6 - 8,4)	5,6 (2,9 - 8,4)	5,8 (1,1 - 10,5)	3,8 (2,3 - 5,3)	0,1 (0,1 - 0,1)	0,2 (0 - 0,4)	305,1 (205,4 - 404,7)
Cauca	4,8 (3,8 - 5,7)	2,5 (1,9 - 3,1)	2,2 (1,3 - 3)	2,8 (1,8 - 3,7)	84,8 (65,2 - 104,4)	12,7 (11 - 14,4)	20,8 (16,6 - 25,1)	0,5 (0,4 - 0,7)	229,6 (150,8 - 308,5)	0,3 (0 - 0,6)	0,3 (0,1 - 0,6)	1,7 (1,3 - 2,1)	4 (3,5 - 4,5)	4,4 (1,4 - 7,3)	1,7 (0,2 - 3,1)	2,7 (1,7 - 3,8)	1 (0,1 - 1,9)	0,3 (0,1 - 0,5)	377,2 (261,1 - 492,9)
Cesar	4,9 (4,1 - 5,8)	1,9 (1,5 - 2,4)	2,7 (1,6 - 3,8)	1,8 (1,4 - 2,2)	114,5 (83,2 - 145,8)	23,8 (20,6 - 27)	23,5 (18,4 - 28,6)	0,7 (0,5 - 1)	216,8 (145,9 - 287,6)	0,2 (0,1 - 0,3)	0,3 (0,1 - 0,5)	3,9 (2,8 - 5)	5 (4,3 - 5,6)	11,8 (3 - 20,6)	5,1 (0,6 - 9,6)	7,1 (4,1 - 10,2)	1 (0,4 - 1,6)	0,3 (0,1 - 0,6)	425,4 (292,8 - 558,2)
Chocó	1,5 (1,2 - 1,9)	0,2 (0,2 - 0,2)	1,8 (1,4 - 2,2)	1,1 (0,6 - 1,5)	50,6 (35,3 - 65,9)	13,4 (10,6 - 16,2)	8,3 (6,2 - 10,3)	0,1 (0,1 - 0,1)	437,6 (279,6 - 595,7)	0 (0 - 0)	0,1 (0,1 - 0,1)	0,9 (0,7 - 1,1)	3,6 (3 - 4,2)	2,5 (1,2 - 3,9)	2,8 (0,7 - 4,9)	3 (1,7 - 4,2)	0,1 (-0,4 - 0,6)	0,1 (0 - 0,1)	527,7 (342,3 - 713,1)
Córdoba	2,4 (1,9 - 2,8)	0,7 (0,5 - 0,8)	1,7 (0,9 - 2,5)	1,5 (1,2 - 1,9)	108 (86,8 - 129,1)	46,5 (40,7 - 52,4)	32,9 (27,3 - 38,4)	1,5 (1 - 2,1)	225,9 (156,7 - 295)	0,2 (0,1 - 0,3)	0,2 (0,1 - 0,3)	2,1 (1,3 - 3)	7,6 (6 - 9,2)	9,8 (3,8 - 15,8)	5,9 (1,3 - 10,5)	11,7 (5,6 - 17,8)	1,4 (0,6 - 2,1)	0,6 (0,3 - 1)	460,7 (336,2 - 585,1)
Cundinamarca	4,5 (3,8 - 5,3)	2 (1,7 - 2,4)	2,9 (1,7 - 4,1)	0,9 (0,6 - 1,2)	122,4 (84,5 - 160,2)	23,7 (20 - 27,3)	21,5 (18 - 24,9)	0,7 (0,5 - 1)	210,9 (138,6 - 283,3)	0,5 (0,1 - 1)	0,5 (0,1 - 0,8)	2,2 (1,5 - 2,8)	8,6 (7,6 - 9,7)	5,4 (2,2 - 8,6)	2,4 (0,3 - 4,5)	3,7 (2,1 - 5,2)	0,8 (0,2 - 1,3)	0,3 (0,1 - 0,4)	413,8 (283,7 - 544)
Guainía	2,2 (-1,8 - 6,2)	0 (0 - 0)	1,9 (1,9 - 1,9)	0 (0 - 0)	39,5 (22,6 - 56,5)	6,6 (6 - 7,2)	10,6 (7 - 14,3)	0 (0 - 0)	48,4 (29,7 - 67)	0,4 (0 - 0)	0 (0 - 0)	4,4 (2,9 - 5,9)	1,7 (1,1 - 2,4)	3,5 (2,6 - 4,5)	1 (0,3 - 1,6)	0,9 (0,9 - 0,9)	0 (0 - 0)	0 (0 - 0)	121,1 (73,2 - 168,4)
Guaviare	4,3 (2,2 - 6,4)	0 (0 - 0)	4 (2,8 - 5,2)	1,1 (-0,5 - 2,8)	53,4 (34,4 - 72,5)	20,7 (18,3 - 23,1)	10,9 (7,8 - 14)	1,4 (1 - 1,7)	101,3 (69,4 - 133,2)	0,5 (0,5 - 0,5)	0,5 (0,5 - 0,5)	1,8 (1 - 2,6)	9,5 (8 - 11)	5,9 (1,5 - 10,4)	1 (0,1 - 2)	1,5 (1,5 - 1,5)	1,3 (1,3 - 1,3)	0,3 (-0,1 - 0,7)	219,4 (149,7 - 289,4)
Huila	2,8 (2,3 - 3,3)	1 (0,8 - 1,2)	8,6 (4,1 - 13,2)	1,1 (0,8 - 1,4)	84 (54,5 - 113,5)	27,2 (22,6 - 31,9)	18,5 (14,4 - 22,5)	0,9 (0,6 - 1,2)	177,9 (128,2 - 227,6)	0,4 (0 - 0,8)	0,2 (0 - 0,3)	1,4 (1 - 1,9)	7,4 (6,5 - 8,3)	5,1 (1,7 - 8,5)	2,6 (0,5 - 4,6)	2 (1,3 - 2,8)	0,6 (0,3 - 0,8)	0,2 (0,1 - 0,4)	341,9 (239,7 - 444,3)
La Guajira	2,9 (2,3 - 3,4)	1,1 (0,6 - 1,6)	0,9 (0,6 - 1,1)	0,4 (0,2 - 0,6)	57,5 (41,3 - 73,7)	16,3 (13,6 - 19)	13,7 (11 - 16,4)	0,1 (0,1 - 0,2)	113,6 (75,2 - 151,9)	0,1 (0 - 0,2)	0,2 (0,1 - 0,4)	1 (0,6 - 1,4)	7,9 (5,8 - 9,9)	9,7 (3,8 - 15,7)	4,1 (0,8 - 7,5)	3,9 (2,2 - 5,6)	0,5 (0,2 - 0,8)	0,3 (0,1 - 0,5)	234,3 (158,7 - 309,8)

Department	Mental and behavioral disorders due to alcohol use	Mental and behavioral disorders due to opioids	Mental and behavioral disorders due to cannabinoids	Mental and behavioral disorders due to cocaine	Schizophrenia	Bipolar disorder	Depression	Dysthymia	Anxiety	Bulimia	Anorexia	Conduct disorders in adults	Intellectual disability	Autism	ADHD	Conduct disorders in childhood and adolescence	Depression in childhood and adolescence	Anxiety in childhood and adolescence	Total
Magdalena	2,2 (1,9 - 2,5)	1,2 (1 - 1,4)	1,2 (0,9 - 1,6)	1,1 (0,8 - 1,4)	224,2 (157,3 - 291,1)	114 (91,6 - 136,4)	22,2 (18 - 26,4)	0,6 (0,4 - 0,7)	184,2 (130,6 - 237,9)	0,2 (0,1 - 0,3)	0,2 (0,1 - 0,3)	1,9 (1,4 - 2,5)	4,5 (3,9 - 5,1)	16,7 (5,9 - 27,6)	2,7 (0,6 - 4,7)	5,9 (3,7 - 8,1)	0,6 (0,3 - 1)	0,3 (0,1 - 0,6)	584 (418,4 - 749,7)
Meta	2,9 (2,4 - 3,4)	1,7 (1,3 - 2,1)	2,1 (1,2 - 3,1)	0,5 (0,4 - 0,7)	85,4 (53,4 - 117,3)	35,6 (29,5 - 41,8)	18,6 (15,2 - 22,1)	0,9 (0,7 - 1,1)	211 (145 - 277)	0,4 (0,2 - 0,7)	0,5 (0,2 - 0,8)	2,2 (1,5 - 2,8)	10,8 (9 - 12,6)	6,4 (2,3 - 10,5)	2,3 (0,2 - 4,3)	4,7 (2,2 - 7,3)	0,9 (0,1 - 1,7)	0,3 (0,1 - 0,5)	387,2 (264,8 - 509,8)
Nariño	6,9 (5,2 - 8,7)	1 (0,8 - 1,2)	3,3 (1,5 - 5,1)	3 (1,7 - 4,3)	133,4 (92,8 - 174)	12,4 (10,4 - 14,5)	25,2 (20 - 30,3)	0,3 (0,2 - 0,5)	152 (99,8 - 204,2)	0,2 (-0,1 - 0,4)	0,3 (0,1 - 0,5)	1,2 (0,7 - 1,7)	4,6 (3,9 - 5,2)	2,4 (1,2 - 3,5)	2,8 (0,6 - 4,9)	1,6 (1,2 - 2,1)	0,2 (0,1 - 0,3)	0,2 (0,1 - 0,4)	350,9 (240,1 - 461,8)
Norte de Santander	4,6 (4 - 5,3)	37 (13,3 - 60,7)	5,6 (2,6 - 8,5)	9,5 (4,4 - 14,6)	51,4 (36,4 - 66,5)	66,2 (54,9 - 77,5)	22 (18,3 - 25,8)	1,5 (0,9 - 2,1)	236,4 (159,3 - 313,5)	0,3 (0 - 0,5)	0,3 (0,1 - 0,5)	0,9 (0,7 - 1,1)	6,3 (5,4 - 7,1)	14 (5,8 - 22,2)	4,9 (0,9 - 8,8)	4,2 (2,6 - 5,8)	0,8 (0,4 - 1,3)	0,3 (0,1 - 0,5)	466,3 (310,2 - 622,4)
Putumayo	6,1 (4,4 - 7,8)	0,2 (0 - 0)	4,3 (2,3 - 6,3)	3,1 (1,4 - 4,8)	34,7 (25,2 - 44,1)	15,5 (13,3 - 17,7)	15,9 (12,1 - 19,7)	0,1 (0,1 - 0,1)	109,5 (74,7 - 144,4)	0,3 (0,1 - 0,5)	0,2 (0,2 - 0,2)	0,6 (0,3 - 0,8)	2,6 (2,2 - 3)	2,5 (1,1 - 3,8)	0,8 (0 - 1,7)	3,1 (1,6 - 4,5)	0,3 (0 - 0,5)	0,2 (0 - 0,3)	199,9 (139,1 - 260,1)
Quindio	10,6 (8,5 - 12,7)	60,1 (20,4 - 99,7)	17,8 (8,1 - 27,5)	55,1 (28,1 - 82)	140,9 (111,3 - 170,5)	132,4 (113,1 - 151,8)	323,6 (257,6 - 389,7)	1,9 (1,2 - 2,5)	527,1 (365,6 - 688,7)	0,5 (0,1 - 1)	0,9 (0,1 - 1,7)	6,6 (4,1 - 9,2)	17,1 (14,7 - 19,4)	13,9 (6,9 - 20,8)	8 (0,9 - 15)	8,2 (5,1 - 11,2)	4,3 (1,1 - 7,5)	0,4 (-0,1 - 0,8)	1329,3 (946,7 - 1711,8)
Risaralda	5,6 (4,6 - 6,6)	32,7 (15,1 - 50,4)	11,6 (6,1 - 17)	12,9 (7,8 - 18)	75,5 (58,1 - 92,9)	112,9 (93,8 - 132)	110,4 (89,2 - 131,6)	5,7 (3,6 - 7,8)	623,8 (416,4 - 831,1)	0,4 (0,2 - 0,7)	0,9 (0,1 - 1,7)	4 (2,5 - 5,5)	13,4 (11,4 - 15,5)	16,1 (5,1 - 27,2)	7,8 (0,9 - 14,6)	4,6 (3,1 - 6,2)	1,3 (-0,1 - 2,8)	0,5 (0,2 - 0,8)	1040,3 (718,4 - 1362,2)
Santander	8,8 (6,5 - 11,1)	2,9 (2,2 - 3,6)	6,2 (3,3 - 9,1)	4,2 (2,5 - 5,9)	131,6 (100,1 - 163,1)	74,3 (62,9 - 85,7)	123,1 (101,7 - 144,6)	2,4 (1,8 - 3,1)	353,1 (236,5 - 469,7)	0,6 (0,2 - 1)	1 (0,2 - 1,8)	3,4 (2,5 - 4,2)	10,8 (9,5 - 12)	16,2 (5,7 - 26,7)	9,1 (1,3 - 16,8)	6,1 (3,7 - 8,5)	2,7 (0,7 - 4,7)	0,6 (0,3 - 0,9)	757 (541,4 - 972,5)
Sucre	19,9 (11,6 - 28,2)	1 (0,8 - 1,1)	1,8 (0,9 - 2,6)	1,4 (1 - 1,8)	74,5 (53 - 96)	32,5 (27,9 - 37)	43,1 (33,4 - 52,8)	0,7 (0,3 - 1,1)	331,6 (224,5 - 438,6)	0,2 (0,1 - 0,3)	0,1 (0,1 - 0,2)	1,5 (1 - 2,1)	11,9 (9,4 - 14,5)	11,7 (4,6 - 18,7)	8,8 (1,3 - 16,3)	8,5 (5,1 - 12)	0,9 (0,3 - 1,6)	0,5 (0,1 - 0,8)	550,7 (375,3 - 725,8)
Tolima	3,8 (3,1 - 4,6)	2 (1,6 - 2,4)	4,1 (2,5 - 5,7)	1,2 (0,8 - 1,6)	116 (78,8 - 153,2)	53,1 (43,9 - 62,4)	62,1 (51,2 - 73)	2,3 (1,1 - 3,5)	342,9 (238,3 - 447,4)	0,5 (0,1 - 0,8)	0,7 (0,2 - 1,2)	2,7 (1,6 - 3,7)	10,2 (8,7 - 11,6)	10,4 (4,5 - 16,4)	2,2 (0,4 - 4,1)	3,5 (2,2 - 4,8)	3,7 (0,4 - 7)	0,5 (0,2 - 0,8)	621,9 (439,7 - 804,2)
Valle del Cauca	2,6 (2,1 - 3,1)	2,7 (1,5 - 3,8)	2,4 (1,4 - 3,4)	2,2 (1,6 - 2,8)	108,4 (79 - 137,9)	32,7 (28,1 - 37,3)	54,2 (43 - 65,4)	1,3 (0,9 - 1,7)	397,4 (268,2 - 526,7)	0,4 (0,1 - 0,8)	0,9 (0,2 - 1,5)	2,8 (1,6 - 4)	7,1 (6,2 - 8)	13,6 (6,2 - 21,1)	4,6 (0,8 - 8,3)	4,2 (2,6 - 5,9)	1,6 (0,3 - 3)	0,6 (0,2 - 0,9)	639,7 (443,9 - 835,5)
Vaupés	3,5 (2,5 - 4,5)	0 (0 - 0)	1,8 (0,3 - 3,4)	1,1 (0 - 0)	8,5 (3,1 - 14)	8,2 (6,2 - 10,3)	24,4 (17,7 - 31,2)	0,3 (0 - 0)	41,9 (29 - 54,7)	0,5 (0 - 0)	0 (0 - 0)	1,1 (-1,2 - 3,3)	4,4 (3,1 - 5,8)	1,9 (-0,1 - 3,9)	0,6 (-0,1 - 1,3)	3,2 (1,2 - 5,1)	0 (0 - 0)	0 (0 - 0)	101,4 (61,6 - 137,6)
Vichada	1,9 (1,1 - 2,7)	0 (0 - 0)	1,3 (-0,1 - 2,7)	0 (0 - 0)	18 (13,1 - 23)	8,1 (6,8 - 9,4)	3,4 (2,1 - 4,7)	4,1 (3 - 5,1)	30,6 (21,3 - 40)	0 (0 - 0)	0 (0 - 0)	2,4 (1,5 - 3,3)	1,8 (1 - 2,6)	0,5 (0,5 - 0,5)	1,6 (0,8 - 2,4)	1,2 (-0,1 - 2,5)	0,2 (0 - 0)	0 (0 - 0)	75,2 (51,1 - 98,9)
