# Supplementary material for: Burden of Mental and Behavioral Disorders in Colombia, 2022: A Subnational Analysis Based on Disability-Adjusted Life Years
Source: Int J Environ Res Public Health. 2025 Dec 12;22(12):1854. doi: 10.3390/ijerph22121854 (PMC12733028; doi:10.3390/ijerph22121854)
Supplement: Supplementary file 1 [file ijerph-22-01854-s001.zip › Table S6.pdf]

Table S6. DALY rate (Disability-Adjusted Life Years) by disorders, by department, Colombia 2022.

Department	Mental and behavioral disorders due to alcohol use	Mental and behavioral disorders due to opioids	Mental and behavioral disorders due to cannabinoids	Mental and behavioral disorders due to cocaine	Schizophrenia	Bipolar disorder	Depression	Dysthymia	Anxiety	Bulimia	Anorexia	Conduct disorders in adults	Intellectual disability	Autism	ADHD	Conduct disorders in childhood and adolescence	Depression in childhood and adolescence	Anxiety in childhood and adolescence	Total
Amazonas	56,3(-30,2 - 142,9)	0,8(0 - 0)	17,8(2,6 - 33,1)	13,9(7,8 - 19,9)	35,3(21,7 - 49)	6,4(5,9 - 6,8)	21,4(15,8 - 27)	0(0 - 0)	76,2(47 - 105,3)	0,3(0 - 0)	0(0 - 0)	6(3,4 - 8,7)	8,3(6,9 - 9,7)	5,9(2,8 - 9,1)	1(-0,4 - 2,4)	3,7(2,7 - 4,7)	0,6(0,6 - 0,6)	0(0 - 0)	254,1(86,7 - 419,3)
Antioquia	16,7(11,5 - 21,9)	7,6(4,7 - 10,6)	9,3(4,7 - 13,9)	14,2(8,1 - 20,3)	67,4(43,6 - 91,2)	66(54,4 - 77,6)	45,6(37 - 54,2)	1,4(1 - 1,8)	298,5(203,8 - 393,2)	0,5(0,2 - 0,8)	3(0,1 - 1,5)	4,4(2,6 - 6,2)	8,4(7,3 - 9,5)	8,9(2,8 - 15)	5,1(0,7 - 9,5)	4,8(2 - 7,6)	0,7(0,4 - 1)	0,2(0,1 - 0,4)	562,8(385 - 736,3)
Arauca	3,6(3,1 - 4,1)	3,7(2,1 - 5,4)	1,9(1,1 - 2,6)	1(0 - 2)	83(61,9 - 104)	31,9(27,2 - 36,6)	20,2(16,1 - 24,3)	2,2(1,6 - 2,8)	271,1(181,9 - 360,2)	0,1(0,1 - 0,1)	0,3(0 - 0,6)	2(1,2 - 2,7)	4,9(4,1 - 5,6)	6,7(3,4 - 10,1)	3,7(0,4 - 6,9)	7,8(4,2 - 11,5)	0,9(0 - 1,7)	0,2(0,1 - 0,4)	445,1(308,5 - 581,8)
Archipiélago de San Andrés, Providencia y Santa Catalina	7,2(5,5 - 8,9)	1,1(0 - 0)	4(2,9 - 5,1)	3,3(1,6 - 4,9)	140(113,7 - 166,2)	60(51,4 - 68,7)	24,4(18,4 - 30,4)	1,4(0,8 - 2)	258(186 - 330,1)	0(0 - 0)	0(0 - 0)	2,7(1,2 - 4,2)	8,8(7,3 - 10,3)	29,8(8,6 - 51)	12,2(0,9 - 23,5)	8,9(5,5 - 12,3)	0,8(0,8 - 0,8)	0(0 - 0)	562,6(404,4 - 718,5)
Atlántico	4,7(2,1 - 7,4)	1,9(1,5 - 2,3)	2(1,2 - 2,9)	2(1,5 - 2,4)	119,8(81,8 - 157,8)	44,2(37,8 - 50,7)	54,9(45,7 - 64)	0,3(0,2 - 0,4)	367,9(250,3 - 485,4)	0,3(0 - 0,5)	0,4(0,2 - 0,6)	2,4(1,3 - 3,6)	11,8(9,5 - 14,2)	40(11,3 - 68,7)	14,7(2,2 - 27,3)	13,1(7,7 - 18,6)	1,3(0,2 - 2,3)	0,5(0,2 - 0,8)	682,3(454,9 - 909,8)
Bogotá, D.C.	11,7(9,1 - 14,3)	3,7(2,9 - 4,5)	5,5(3,3 - 7,6)	2,2(1,6 - 2,8)	116,7(80 - 153,4)	44,6(36,9 - 52,2)	50,9(43,2 - 58,6)	2,2(1,5 - 2,9)	324,6(215 - 434,2)	1(0,2 - 1,7)	1,6(0,4 - 2,7)	4,7(2,8 - 6,6)	12(10,4 - 13,5)	12,4(5,3 - 19,5)	5,8(0,7 - 10,9)	5,1(3,2 - 7,1)	1,8(0,3 - 3,2)	0,4(0,1 - 0,7)	606,8(416,9 - 796,6)
Bolívar	6,6(2,5 - 10,7)	1,4(1,2 - 1,7)	3,5(1,8 - 5,1)	4,1(2,5 - 5,7)	138,1(98,2 - 178)	82,4(65,3 - 99,5)	85,8(64,7 - 106,8)	0,4(0,2 - 0,6)	348(236,9 - 459,2)	0,2(0,1 - 0,3)	0,3(0,1 - 0,6)	3,5(2 - 5)	14,1(10,5 - 17,8)	29(8,5 - 49,4)	11,6(3,2 - 20)	14,9(8,7 - 21,1)	0,7(0,2 - 1,2)	0,4(0,1 - 0,7)	745(506,8 - 983,3)
Boyacá	22(7,5 - 36,4)	2,1(1,8 - 2,3)	2,7(2 - 3,5)	1,3(0,9 - 1,7)	96,7(70,2 - 123,3)	54,8(46,6 - 63,1)	47(37 - 56,9)	1,4(0,9 - 1,9)	364,2(240,2 - 488,2)	0,8(0,3 - 1,3)	0,5(0 - 1,1)	3,1(2,3 - 3,8)	10,6(9,3 - 12)	7(2,7 - 11,4)	2,6(0,5 - 4,8)	3,4(1,9 - 4,8)	2,1(0,2 - 4,1)	0,4(0,1 - 0,7)	622,8(424,4 - 821,2)
Caldas	15,9(10,1 - 21,6)	2,8(2,1 - 3,4)	12,7(6,3 - 19,2)	4,3(2,9 - 5,8)	80,3(56,6 - 104)	161,5(137,1 - 186)	139,3(114,7 - 164)	2(1,3 - 2,8)	534,2(354,6 - 713,8)	0,2(-0,1 - 0,5)	0,7(0 - 1,3)	5,9(3,6 - 8,2)	14,2(11,9 - 16,5)	10,8(5,2 - 16,4)	11,6(3,5 - 19,8)	7(4,7 - 9,3)	2,5(0,1 - 4,8)	0,4(0 - 0,8)	1006,4(714,7 - 1298)
Caquetá	4(3,4 - 4,7)	2(1,5 - 2,4)	15(8 - 21,9)	4,1(2,9 - 5,4)	28,4(20,7 - 36,1)	85,2(69,4 - 101,1)	27,5(20,3 - 34,7)	0,3(0,2 - 0,5)	201,9(131,4 - 272,4)	0,4(0,2 - 0,5)	0,4(0,2 - 0,6)	1,9(1 - 2,7)	11,6(9,4 - 13,8)	5,8(0,2 - 11,5)	2,6(0,4 - 4,9)	1,2(0,9 - 1,5)	0,4(0,2 - 0,6)	0,4(0 - 0,8)	393,1(270,3 - 515,9)
Casanare	1,5(1,3 - 1,8)	0,9(0,9 - 0,9)	2(1 - 2,9)	0,9(0,7 - 1)	37(26,3 - 47,7)	34,3(28 - 40,7)	6,6(5 - 8,1)	0,2(-0,1 - 0,4)	196,3(128,9 - 263,7)	0(0 - 0)	0,1(-0,5 - 0,8)	2,5(1,6 - 3,5)	7,2(6 - 8,4)	5,6(2,9 - 8,4)	5,8(1,1 - 10,5)	3,8(2,3 - 5,3)	0,1(0,1 - 0,1)	0,2(0 - 0,4)	305,1(205,4 - 404,7)
Cauca	8,1(3,4 - 12,8)	2,5(1,9 - 3,1)	2,2(1,3 - 3)	2,8(1,8 - 3,7)	84,8(65,2 - 104,4)	12,7(11 - 14,4)	20,8(16,6 - 25,1)	0,5(0,4 - 0,7)	229,6(150,8 - 308,5)	0,3(0 - 0,6)	0,3(0,1 - 0,6)	1,7(1,3 - 2,1)	4(3,5 - 4,5)	4,4(1,4 - 7,3)	1,7(0,2 - 3,1)	2,7(1,7 - 3,8)	1(0,1 - 1,9)	0,3(0,1 - 0,5)	380,5(260,8 - 500,1)
Cesar	7,2(2,7 - 11,7)	1,9(1,5 - 2,4)	2,7(1,6 - 3,8)	1,8(1,4 - 2,2)	117,2(85,7 - 148,6)	23,8(20,6 - 27)	23,5(18,4 - 28,6)	0,7(0,5 - 1)	216,8(145,9 - 287,6)	0,2(0,1 - 0,3)	0,3(0,1 - 0,5)	3,9(2,8 - 5)	5(4,3 - 5,6)	11,8(3 - 20,6)	5,1(0,6 - 9,6)	7,1(4,1 - 10,2)	1(0,4 - 1,6)	0,3(0,1 - 0,6)	430,4(293,8 - 566,9)
Chocó	13,7(-3,7 - 31,1)	0,2(0,2 - 0,2)	1,8(1,4 - 2,2)	1,1(0,6 - 1,5)	50,6(35,3 - 65,9)	13,4(10,6 - 16,2)	8,3(6,2 - 10,3)	0,1(0,1 - 0,1)	437,6(279,6 - 595,7)	0(0 - 0)	0,1(0,1 - 0,1)	0,9(0,7 - 1,1)	3,6(3 - 4,2)	2,5(1,2 - 3,9)	2,8(0,7 - 4,9)	3(1,7 - 4,2)	0,1(-0,4 - 0,6)	0,1(0 - 0,1)	539,8(337,4 - 742,3)
Córdoba	2,4(1,9 - 2,8)	0,7(0,5 - 0,8)	1,7(0,9 - 2,5)	1,5(1,2 - 1,9)	108(86,8 - 129,1)	46,5(40,7 - 52,4)	32,9(27,3 - 38,4)	1,5(1 - 2,1)	225,9(156,7 - 295)	0,2(0,1 - 0,3)	0,2(0,1 - 0,3)	2,1(1,3 - 3)	7,6(6 - 9,2)	9,8(3,8 - 15,8)	5,9(1,3 - 10,5)	11,7(5,6 - 17,8)	1,4(0,6 - 2,1)	0,6(0,3 - 1)	460,7(336,2 - 585,2)
Cundinamarca	7,3(3,8 - 10,8)	2(1,7 - 2,4)	2,9(1,7 - 4,1)	0,9(0,6 - 1,2)	122,4(84,5 - 160,2)	23,7(20 - 27,3)	21,5(18 - 24,9)	0,7(0,5 - 1)	210,9(138,6 - 283,3)	0,5(0,1 - 1)	0,5(0,1 - 0,8)	2,2(1,5 - 2,8)	8,6(7,6 - 9,7)	5,4(2,2 - 8,6)	2,4(0,3 - 4,5)	3,7(2,1 - 5,2)	0,8(0,2 - 1,3)	0,3(0,1 - 0,4)	416,6(283,7 - 549,5)
Guainía	2,2(-1,8 - 6,2)	0(0 - 0)	1,9(1,9 - 1,9)	0(0 - 0)	39,5(22,6 - 56,5)	6,6(6 - 7,2)	10,6(7 - 14,3)	0(0 - 0)	48,4(29,7 - 67)	0,4(0 - 0)	0(0 - 0)	4,4(2,9 - 5,9)	1,7(1,1 - 2,4)	3,5(2,6 - 4,5)	1(0,3 - 1,6)	0,9(0,9 - 0,9)	0(0 - 0)	0(0 - 0)	121,2(73,3 - 168,4)
Guaviare	4,3(2,2 - 6,4)	0(0 - 0)	4(2,8 - 5,2)	1,1(-0,5 - 2,8)	53,4(34,4 - 72,5)	20,7(18,3 - 23,1)	10,9(7,8 - 14)	1,4(1 - 1,7)	101,3(69,4 - 133,2)	0,5(0,5 - 0,5)	0,5(0,5 - 0,5)	1,8(1 - 2,6)	9,5(8 - 11)	5,9(1,5 - 10,4)	1(0,1 - 2)	1,5(1,5 - 1,5)	1,3(1,3 - 1,3)	0,3(-0,1 - 0,7)	219,5(149,7 - 289,3)
Huila	4,3(1,4 - 7,1)	1(0,8 - 1,2)	8,6(4,1 - 13,2)	1,1(0,8 - 1,4)	84(54,5 - 113,5)	27,2(22,6 - 31,9)	18,5(14,4 - 22,5)	0,9(0,6 - 1,2)	177,9(128,2 - 227,6)	0,4(0 - 0,8)	0,2(0 - 0,3)	1,4(1 - 1,9)	7,4(6,5 - 8,3)	5,1(1,7 - 8,5)	2,6(0,5 - 4,6)	2(1,3 - 2,8)	0,6(0,3 - 0,8)	0,2(0,1 - 0,4)	343,4(238,7 - 448)

Department	Mental and behavioral disorders due to alcohol use	Mental and behavioral disorders due to opioids	Mental and behavioral disorders due to cannabinoids	Mental and behavioral disorders due to cocaine	Schizophrenia	Bipolar disorder	Depression	Dysthymia	Anxiety	Bulimia	Anorexia	Conduct disorders in adults	Intellectual disability	Autism	ADHD	Conduct disorders in childhood and adolescence	Depression in childhood and adolescence	Anxiety in childhood and adolescence	Total
La Guajira	8,9(0,4 - 17,4)	1,1(0,6 - 1,6)	0,9(0,6 - 1,1)	0,4(0,2 - 0,6)	57,5(41,3 - 73,7)	16,3(13,6 - 19)	13,7(11 - 16,4)	0,1(0,1 - 0,2)	113,6(75,2 - 151,9)	0,1(0 - 0,2)	0,2(0,1 - 0,4)	1(0,6 - 1,4)	7,9(5,8 - 9,9)	9,7(3,8 - 15,7)	4,1(0,8 - 7,5)	3,9(2,2 - 5,6)	0,5(0,2 - 0,8)	0,3(0,1 - 0,5)	240,3(156,9 - 323,7)
Magdalena	12(0,3 - 23,7)	1,2(1 - 1,4)	1,2(0,9 - 1,6)	1,1(0,8 - 1,4)	224,2(157,3 - 291,1)	114(91,6 - 136,4)	22,2(18 - 26,4)	0,6(0,4 - 0,7)	184,2(130,6 - 237,9)	0,2(0,1 - 0,3)	0,2(0,1 - 0,3)	1,9(1,4 - 2,5)	4,5(3,9 - 5,1)	16,7(5,9 - 27,6)	2,7(0,6 - 4,7)	5,9(3,7 - 8,1)	0,6(0,3 - 1)	0,3(0,1 - 0,6)	593,8(416,8 - 770,8)
Meta	2,9(2,4 - 3,4)	1,7(1,3 - 2,1)	2,1(1,2 - 3,1)	0,5(0,4 - 0,7)	85,4(53,4 - 117,3)	35,6(29,5 - 41,8)	18,6(15,2 - 22,1)	0,9(0,7 - 1,1)	211(145 - 277)	0,4(0,2 - 0,7)	0,5(0,2 - 0,8)	2,2(1,5 - 2,8)	10,8(9 - 12,6)	6,4(2,3 - 10,5)	2,3(0,2 - 4,3)	4,7(2,2 - 7,3)	0,9(0,1 - 1,7)	0,3(0,1 - 0,5)	387,3(264,9 - 509,8)
Nariño	9,1(4,7 - 13,4)	1(0,8 - 1,2)	3,3(1,5 - 5,1)	3(1,7 - 4,3)	136,7(95,9 - 177,4)	12,4(10,4 - 14,5)	25,2(20 - 30,3)	0,3(0,2 - 0,5)	152(99,8 - 204,2)	0,2(-0,1 - 0,4)	0,3(0,1 - 0,5)	1,2(0,7 - 1,7)	4,6(3,9 - 5,2)	2,4(1,2 - 3,5)	2,8(0,6 - 4,9)	1,6(1,2 - 2,1)	0,2(0,1 - 0,3)	0,2(0,1 - 0,4)	356,3(242,8 - 469,9)
Norte de Santander	5,4(3,8 - 6,9)	37(13,3 - 60,7)	5,6(2,6 - 8,5)	9,5(4,4 - 14,6)	51,4(36,4 - 66,5)	66,2(54,9 - 77,5)	22(18,3 - 25,8)	1,5(0,9 - 2,1)	236,4(159,3 - 313,5)	0,3(0 - 0,5)	0,3(0,1 - 0,5)	0,9(0,7 - 1,1)	6,3(5,4 - 7,1)	14(5,8 - 22,2)	4,9(0,9 - 8,8)	4,2(2,6 - 5,8)	0,8(0,4 - 1,3)	0,3(0,1 - 0,5)	467(310,1 - 624)
Putumayo	6,1(4,4 - 7,8)	0,2(0 - 0)	4,3(2,3 - 6,3)	3,1(1,4 - 4,8)	34,7(25,2 - 44,1)	15,5(13,3 - 17,7)	15,9(12,1 - 19,7)	0,1(0,1 - 0,1)	109,5(74,7 - 144,4)	0,3(0,1 - 0,5)	0,2(0,2 - 0,2)	0,6(0,3 - 0,8)	2,6(2,2 - 3)	2,5(1,1 - 3,8)	0,8(0 - 1,7)	3,1(1,6 - 4,5)	0,3(0 - 0,5)	0,2(0 - 0,3)	199,8(139 - 260,2)
Quindio	10,6(8,5 - 12,7)	60,1(20,4 - 99,7)	17,8(8,1 - 27,5)	55,1(28,1 - 82)	140,9(111,3 - 170,5)	132,4(113,1 - 151,8)	323,6(257,6 - 389,7)	1,9(1,2 - 2,5)	527,1(365,6 - 688,7)	0,5(0,1 - 1)	0,9(0,1 - 1,7)	6,6(4,1 - 9,2)	17,1(14,7 - 19,4)	13,9(6,9 - 20,8)	8(0,9 - 15)	8,2(5,1 - 11,2)	4,3(1,1 - 7,5)	0,4(-0,1 - 0,8)	1329,3(946,7 - 1712)
Risaralda	13(2 - 24)	32,7(15,1 - 50,4)	11,6(6,1 - 17)	12,9(7,8 - 18)	75,5(58,1 - 92,9)	112,9(93,8 - 132)	110,4(89,2 - 131,6)	5,7(3,6 - 7,8)	623,8(416,4 - 831,1)	0,4(0,2 - 0,7)	0,9(0,1 - 1,7)	4(2,5 - 5,5)	13,4(11,4 - 15,5)	16,1(5,1 - 27,2)	7,8(0,9 - 14,6)	4,6(3,1 - 6,2)	1,3(-0,1 - 2,8)	0,5(0,2 - 0,8)	1047,6(715,7 - 1379,6)
Santander	17,6(10,2 - 25,1)	4,6(1,2 - 8)	6,2(3,3 - 9,1)	4,2(2,5 - 5,9)	131,6(100,1 - 163,1)	74,3(62,9 - 85,7)	123,1(101,7 - 144,6)	2,4(1,8 - 3,1)	353,1(236,5 - 469,7)	0,6(0,2 - 1)	1(0,2 - 1,8)	3,4(2,5 - 4,2)	10,8(9,5 - 12)	16,2(5,7 - 26,7)	9,1(1,3 - 16,8)	6,1(3,7 - 8,5)	2,7(0,7 - 4,7)	0,6(0,3 - 0,9)	767,5(544,2 - 990,9)
Sucre	25(12,3 - 37,8)	1(0,8 - 1,1)	1,8(0,9 - 2,6)	1,4(1 - 1,8)	74,5(53 - 96)	32,5(27,9 - 37)	43,1(33,4 - 52,8)	0,7(0,3 - 1,1)	331,6(224,5 - 438,6)	0,2(0,1 - 0,3)	0,1(0,1 - 0,2)	1,5(1 - 2,1)	11,9(9,4 - 14,5)	11,7(4,6 - 18,7)	8,8(1,3 - 16,3)	8,5(5,1 - 12)	0,9(0,3 - 1,6)	0,5(0,1 - 0,8)	555,8(376,1 - 735,4)
Tolima	4,8(2,9 - 6,7)	2(1,6 - 2,4)	4,1(2,5 - 5,7)	1,2(0,8 - 1,6)	116(78,8 - 153,2)	53,1(43,9 - 62,4)	62,1(51,2 - 73)	2,3(1,1 - 3,5)	342,9(238,3 - 447,4)	0,5(0,1 - 0,8)	0,7(0,2 - 1,2)	2,7(1,6 - 3,7)	10,2(8,7 - 11,6)	10,4(4,5 - 16,4)	2,2(0,4 - 4,1)	3,5(2,2 - 4,8)	3,7(0,4 - 7)	0,5(0,2 - 0,8)	622,8(439,4 - 806,3)
Valle del Cauca	7,4(2,7 - 12,2)	2,7(1,5 - 3,8)	2,4(1,4 - 3,4)	2,2(1,6 - 2,8)	108,4(79 - 137,9)	32,7(28,1 - 37,3)	54,2(43 - 65,4)	1,3(0,9 - 1,7)	397,4(268,2 - 526,7)	0,4(0,1 - 0,8)	0,9(0,2 - 1,5)	2,8(1,6 - 4)	7,1(6,2 - 8)	13,6(6,2 - 21,1)	4,6(0,8 - 8,3)	4,2(2,6 - 5,9)	1,6(0,3 - 3)	0,6(0,2 - 0,9)	644,5(444,4 - 844,5)
Vaupés	3,5(2,5 - 4,5)	0(0 - 0)	1,8(0,3 - 3,4)	1,1(0 - 0)	8,5(3,1 - 14)	8,2(6,2 - 10,3)	24,4(17,7 - 31,2)	0,3(0 - 0)	41,9(29 - 54,7)	0,5(0 - 0)	0(0 - 0)	1,1(-1,2 - 3,3)	4,4(3,1 - 5,8)	1,9(-0,1 - 3,9)	0,6(-0,1 - 1,3)	3,2(1,2 - 5,1)	0(0 - 0)	0(0 - 0)	101,4(61,6 - 137,6)
Vichada	1,9(1,1 - 2,7)	0(0 - 0)	1,3(-0,1 -)	0(0 - 0)	18(13,1 - 23)	8,1(6,8 - 9,4)	3,4(2,1 - 4,7)	4,1(3 - 5,1)	30,6(21,3 - 40)	0(0 - 0)	0(0 - 0)	2,4(1,5 - 3,3)	1,8(1 - 2,6)	0,5(0,5 - 0,5)	1,6(0,8 - 2,4)	1,2(-0,1 - 2,5)	0,2(0 - 0)	0(0 - 0)	75,2(51,1 - 96,1)
